# The impact of absorbed photons on antimicrobial photodynamic efficacy

**DOI:** 10.3389/fmicb.2015.00706

**Published:** 2015-07-15

**Authors:** Fabian Cieplik, Andreas Pummer, Johannes Regensburger, Karl-Anton Hiller, Andreas Späth, Laura Tabenski, Wolfgang Buchalla, Tim Maisch

**Affiliations:** ^1^Department of Conservative Dentistry and Periodontology, University Medical Center RegensburgRegensburg, Germany; ^2^Department of Dermatology, University Medical Center RegensburgRegensburg, Germany; ^3^Department of Organic Chemistry, University of RegensburgRegensburg, Germany

**Keywords:** photodynamic, absorbed photons, biofilm, SAPYR, Methylene Blue, antimicrobial resistance

## Abstract

Due to increasing resistance of pathogens toward standard antimicrobial procedures, alternative approaches that are capable of inactivating pathogens are necessary in support of regular modalities. In this instance, the photodynamic inactivation of bacteria (PIB) may be a promising alternative. For clinical application of PIB it is essential to ensure appropriate comparison of given photosensitizer (PS)-light source systems, which is complicated by distinct absorption and emission characteristics of given PS and their corresponding light sources, respectively. Consequently, in the present study two strategies for adjustment of irradiation parameters were evaluated: (i) matching energy doses applied by respective light sources (common practice) and (ii) by development and application of a formula for adjusting the numbers of photons absorbed by PS upon irradiation by their corresponding light sources. Since according to the photodynamic principle one PS molecule is excited by the absorption of one photon, this formula allows comparison of photodynamic efficacy of distinct PS per excited molecule. In light of this, the antimicrobial photodynamic efficacy of recently developed PS SAPYR was compared to that of clinical standard PS Methylene Blue (MB) regarding inactivation of monospecies biofilms formed by *Enterococcus faecalis* and *Actinomyces naeslundii* whereby evaluating both adjustment strategies. PIB with SAPYR exhibited CFU-reductions of 5.1 log_10_ and 6.5 log_10_ against *E. faecalis* and *A. naeslundii*, respectively, which is declared as a disinfectant efficacy. In contrast, the effect of PIB with MB was smaller when the applied energy dose was adjusted compared to SAPYR (CFU-reductions of 3.4 log_10_ and 4.2 log_10_ against *E. faecalis* and *A. naeslundii*), or there was even no effect at all when the number of absorbed photons was adjusted compared to SAPYR. Since adjusting the numbers of absorbed photons is the more precise and adequate method from a photophysical point of view, this strategy should be considered in further studies when antimicrobial efficacy rates of distinct PS-light source systems are compared.

## Introduction

In a current assessment the Centers for Disease Control and Prevention of the United States of America stated that antibiotic-resistant bacteria are causative for at least two million illnesses and 23,000 deaths per year in the U.S. alone (Centers for Disease Control and Prevention, 2013). Consequently, in September 2014, the White House published a strategy paper where the rise of antibiotic-resistant bacteria was declared as a serious threat to public health and economy. Some of the goals outlined in this strategy paper were to promote judicious use of antibiotics and to accelerate basic and applied research on and development of novel therapeutic tools for combating bacterial resistance (The White House, 2014).

In particular in the field of dentistry, it should be a major goal to moderate the application of systemic antibiotics. For example, it is common practice to deliver Amoxicillin and Metronidazol combined for 1 week in addition to conventional scaling and root planing for treatment of severe forms of chronic periodontitis (Winkelhoff et al., 1996; Zandbergen et al., 2013), which is critically judged more and more nowadays (Preus et al., 2014). Furthermore, dentists are increasingly faced with situations, where they have to deal with refractory progressions of periodontal or endodontic infections caused by antibiotic-resistant pathogens (Al-Ahmad et al., 2014; Rams et al., 2014).

In light of this, a promising alternative may be the photodynamic inactivation of bacteria (PIB). PIB is an antimicrobial approach consisting of the application of a *per se* non-toxic molecule, the so-called photosensitizer (PS), and subsequent irradiation with visible light of an adequate wavelength in the presence of molecular oxygen. The absorption of light by the PS molecule transfers it to its energized triplet state, from which there are two mechanisms to regain its ground state: In type I mechanism, charge is transferred to a substrate or to molecular oxygen with emergence of reactive oxygen species (ROS) like superoxide anions (O_2_−•), free hydroxyl radicals (HO•) and hydrogen peroxide (H_2_O_2_). In contrast, in type II mechanism, energy is transferred directly to molecular oxygen, whereby highly reactive singlet oxygen (^1^O_2_) is generated (Wainwright, 1998; Schweitzer and Schmidt, 2003). The proportion of both PIB mechanisms is unique for each PS with the singlet oxygen quantum yield Φ _Δ_ describing the proportion of type II mechanism (Maisch et al., 2007).

Currently, in dental practice predominantly PS based on a phenothiazinium structure are clinically used like Methylene Blue (MB) (Gursoy et al., 2013). MB has a strong absorption in the red spectral region (λ _abs_ = 600−680 nm) (Felgenträger et al., 2013) and its singlet oxygen quantum yield Φ _Δ_ is 0.52 (Wilkinson et al., 1993). However, results with MB regarding inactivation of biofilms *in vitro* are quite conflicting (Cieplik et al., 2014b) and clinical trials on PIB with MB as a supportive modality for treatment of periodontitis suggest that there are no long-term positive effects (Sgolastra et al., 2013). In contrast, SAPYR has recently been introduced by our group as a new class of PS based on a phenalen-1-one structure with a positively charged pyridinium-methyl substituent and has shown pronounced antimicrobial efficacy against planktonic bacteria (Späth et al., 2014) as well as biofilms (Cieplik et al., 2013). SAPYR absorbs in a range from UV-A to the blue spectral region (λ _abs_ = 360−420 nm) and exhibits a singlet oxygen quantum yield Φ _Δ_ = 0.99, thus reacting virtually exclusively according to type II mechanism (Cieplik et al., 2013; Späth et al., 2014).

For appropriate comparison of antimicrobial photodynamic efficacy rates of given PS-light source systems, it is essential to adjust irradiation parameters, which is complexed by distinct absorption and emission characteristics of given PS and their corresponding light sources. Hereby, it is common practice to match the energy doses applied by the respective light sources. In this instance, emission and absorption characteristics are mostly left without consideration. However, – according to the photodynamic principle – one PS molecule is excited by the absorption of one photon, wherefore the numbers of photons absorbed by PS upon irradiation by their corresponding light sources should be calculated allowing comparison of photodynamic efficacy rates of distinct PS per excited molecule.

Aim of the present study was to evaluate two strategies for adjustment of irradiation parameters: (I) adjusting the energy doses applied by the respective light sources irrespective of spectral properties and (II) by development and application of a formula for calculating the numbers of absorbed photons for each PS upon irradiation by its corresponding light source. Applying these strategies, SAPYR and MB were exemplarily compared regarding inactivation of monospecies biofilms formed by oral key pathogens *E. faecalis* and *A. naeslundii*.

## Materials and methods

### Photosensitizers and light sources

**Figure 1:**
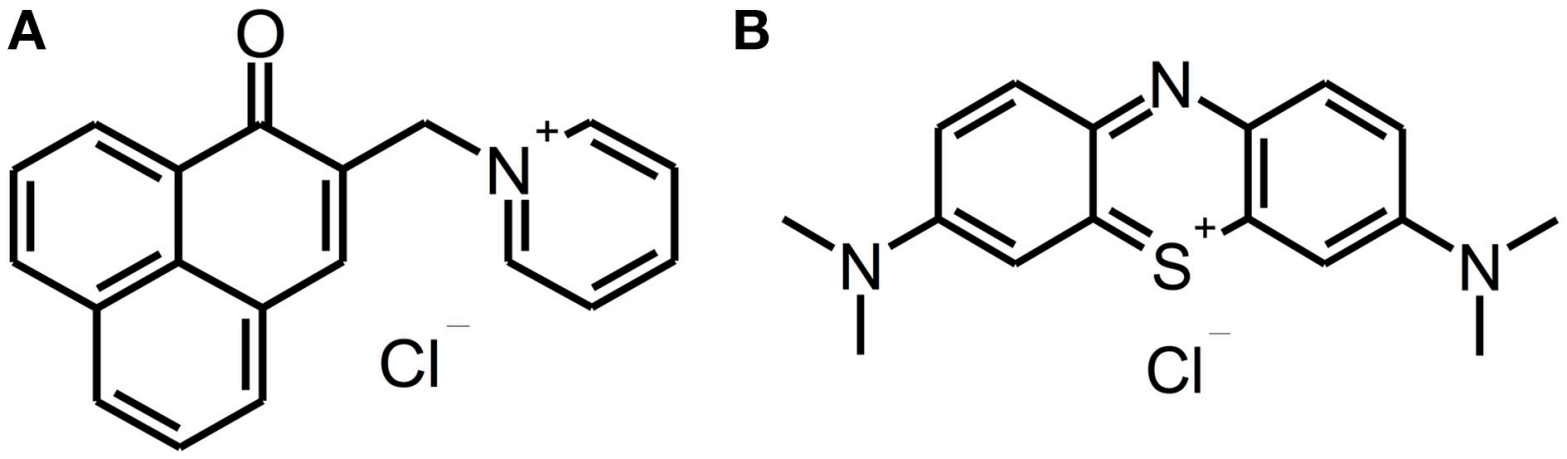
**Chemical structures of SAPYR and Methylene Blue**. Chemical structures of phenalen-1-one PS SAPYR **(A)** and phenothiazinium PS Methylene Blue **(B)**.

The phenalen-1-one PS SAPYR [2-((4-pyridinyl)methyl)-1H-phenalen-1-one chloride; Figure 1A] was synthetized at the Department of Organic Chemistry (University of Regensburg, Germany) with a purity of ≥ 99%, controlled by NMR and HPLC-MS, as described earlier (Cieplik et al., 2013; Späth et al., 2014). The phenothiazinium PS Methylene Blue [3,7-bis(dimethylamino)-phenothiazin-5-ium chloride; Figure 1B] was purchased from Sigma-Aldrich (St. Louis, MO) and used as received (purity ≥ 95%). Absorption spectra of SAPYR and MB were recorded by means of a photosprectrometer (DU 640, Beckman-Coulter, Krefeld, Germany).

**Figure 2:**
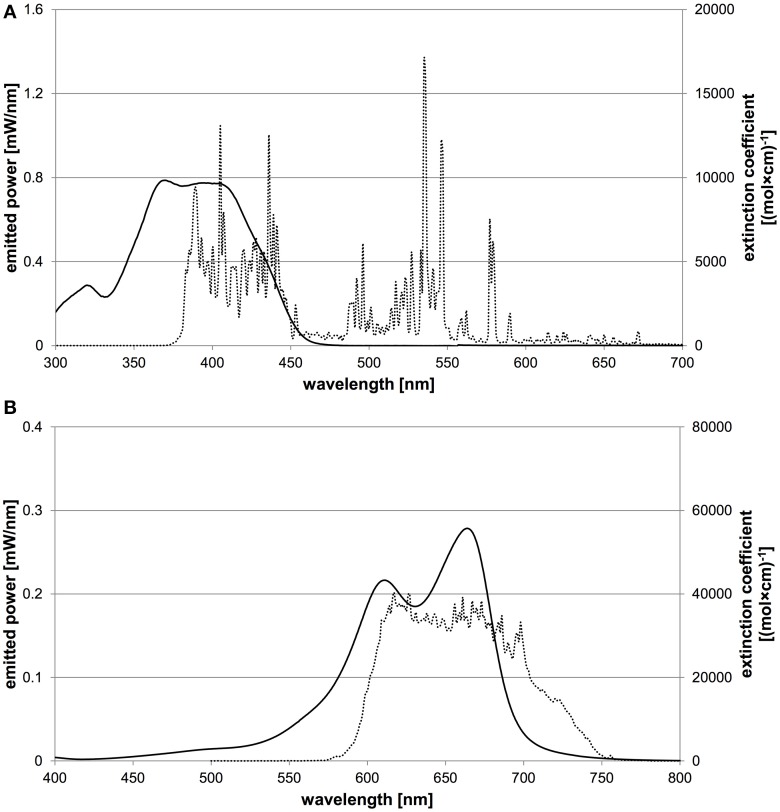
**Characteristic absorption spectra of both PS and emission spectra of corresponding light sources**. The abscissa shows the wavelength in nm, the left ordinate the spectral radiant power of the light source and the right ordinate the extinction coefficient of the PS. **(A)** Characteristic absorption spectrum of SAPYR (solid line) and emission spectrum of Waldmann PIB 3000 (dotted line). **(B)** Characteristic absorption spectrum of MB (solid line) and emission spectrum of Waldmann PDT 1200L (dotted line).

For irradiation of SAPYR, a Waldmann PIB 3000 blue light source (λ _em_ = 380–600 nm) was used, whereas MB was irradiated with a Waldmann PDT 1200L red light source (λ _em_ = 580–750 nm) (both: Waldmann Medizintechnik, Villingen-Schwenningen, Germany). Emission spectra of the light sources were recorded by means of a monochromator with a CCD detection system (SPEX 232, HORIBA Jobin Yvon, Longjumeau Cedex, France). Figure 2 shows the absorption spectra of both PS and the emission spectra of the corresponding light sources.

For PIB experiments, the light intensities obtained from both light sources at the level of the samples were measured with a thermal low-power sensor (Nova 30 A-P-SH, Ophir-Spiricon, North Logan, UT).

### Calculation of irradiation parameters

For calculation of the numbers of absorbed photons, the extinction coefficients of the PS at distinct wavelengths [ε (λ)] and the spectral radiant power emitted by the respective light sources P_em_ (λ) were measured. For determination of the spectral resolved absorption, the concentration of the PS (c, here: 100 μM) and the thickness of the solution (d, here: 1.3 mm) are necessary parameters. Moreover, the universal physical constant of the velocity of light (c_0_) and the Planck constant (h) are required.

$$\begin{array}{l} \text{absorbed photons/ second}=\\ \;\sum_{\lambda }\left(1-10^{-\varepsilon (\lambda )\cdot \text{ c}\cdot \text{ d}}\right)\;\cdot \;{\text{P}}_{\text{em}}(\lambda )\;\cdot \;\frac{\lambda }{{\text{c}}_{0}\;\cdot \text{ h}} \end{array}$$

**Table:** 

ε (λ)	Extinction coefficient [(mol×cm)^−1^]	Measured spectrally resolved by a photospectrometer
P_em_(λ)	Spectral radiant power of the respective light source [mW/nm]	Measured spectrally resolved by a CCD detector system
c	Concentration of the PS [μM]	Here: 100 μM
d	Thickness of the solution [mm]	Here: 1.3 mm
c_0_	Velocity of light [m/s]	299,792,458 m/s
h	Planck constant [J×s]	6.62606957×10^−34^ Js
λ	Wavelength [nm]	

First, irradiation parameters for SAPYR were determined to irradiation with PIB 3000 for 600 s at an irradiance at sample- level of 50 mW/cm^2^, corresponding to an energy dose of 30 J/cm^2^.

In order to compare antimicrobial photodynamic efficacy of SAPYR and MB at corresponding energy dose, irradiation of MB was with PDT 1200L at 50 mW/cm^2^ for 600 s.

For comparing antimicrobial photodynamic efficacy of both PS at matching numbers of absorbed photons, the constants, the experimental data and the measured values were inserted in the formula outlined above, resulting in a number of 1.13 × 10^16^ photons/second absorbed by SAPYR for irradiation with PIB 3000 (irradiance at sample-level: 50 mW/cm^2^). Likewise, a number of 3.75 × 10^16^ photons/second absorbed by MB for irradiation with PDT 1200L (irradiance at sample-level: 20 mW/cm^2^) was calculated. Consequently, in order to adjust the numbers of absorbed photons for both PS, a 3.32 times longer irradiation period was necessary for SAPYR with PIB 3000 compared to MB with PDT 1200L. Therefore, irradiation periods were determined to be 600 s for SAPYR with PIB 3000 (energy dose: 30 J/cm^2^) and 181 s for MB with PDT 1200L (energy dose: 3.6 J/cm^2^).

### Biofilm formation

*Enterococcus faecalis* ATCC 29212 and *Actinomyces naeslundii* T14V were used as model organisms in this study. Monospecies biofilms were cultured, as it has been described earlier (Cieplik et al., 2013). Biofilm formation was in 96-well polystyrene culture plates (Corning Costar®, Corning, NY) with the complete saliva medium (CS) described by Pratten et al. (1998), modified by adding 1% sucrose and applying N_2_ up to O_2_ = 0% in order to ensure anaerobic medium conditions (Tabenski et al., 2014). *E. faecalis* and *A. naeslundii* were incubated under aerobic conditions in Brain Heart Infusion broth (BHI; Sigma-Aldrich, St. Louis, MO) at 37°C on an orbital shaker as overnight-cultures in order to obtain bacteria in the static growth phase. Afterwards, suspensions were harvested by centrifugation (1700 g, 5 min; Megafuge 1.0, Heraeus Sepatech, Osterode, Germany) and re-suspended in sterile phosphate-buffered saline (PBS; Sigma-Aldrich, St. Louis, MO) yielding an optical density (OD) of 0.1, which was measured at 600 nm by means of a photospectrometer (SPECORD 50 PLUS, Analytik Jena, Jena, Germany) and corresponds to a bacterial count of 10^4^ to 10^5^ bacterial cells per ml. Subsequently, bacterial suspensions were pelletized again and re-suspended in CS so that they could be used for biofilm formation. Wells were inoculated with 200 μl of these *E. faecalis* or *A. naeslundii* suspensions and incubated aerobically at 37°C for 72 h, while the medium was substituted every 24 h.

### Confirmation of extracellular polymeric substance (EPS)

The presence of EPS in *A. naeslundii* and *E. faecalis* monospecies biofilms was verified by a lectinsorbent assay (ELLA), based on the manual described by Leriche et al. (2000). Concanavalin A (Con A) was chosen as a lectin, since it binds to the most common monosaccharide-residues (_D_-glucose and _D_-mannose) in the EPS of bacterial biofilms (Sutherland, 1990).

Peroxidase-labeled Con A (Sigma-Aldrich, St. Louis, MO) was diluted in PBS containing 0.05% (vol/vol) Tween 20 (Merck, Darmstadt, Germany) (diluting buffer; DB) obtaining final concentrations of 10.0 and 12.5 μg/ml of Con A. *E. faecalis* and *A. naeslundii* monospecies biofilms were grown as described above in 96-well plates yielding 8 wells in a row colonized with biofilm for each sample. After 72 h, medium was removed and biofilms were washed carefully with PBS to remove non-adherent bacteria. 200 μl of the Con A solution at concentrations of 10.0 μg/ml (*A. naeslundii*, *E. faecalis*) or 12.5 μg/ml (*E. faecalis*), respectively, were added to the first of the eight wells in a row and 100 μl DB to the remaining wells. Consequently, serial half dilutions were performed by transferring 100 μl from the previous to the proximate well. Wells incubated with CS medium for 72 h were submitted to the same procedure and used as a blank. Well plates were incubated in the dark for 60 min at room temperature to allow lectin-binding. Afterwards, peroxidase-labeled Con A solutions were removed from the wells by inverting the plates. Subsequently, the biofilms were washed triply with DB to remove unbound lectin and peroxidase-labeled Con A was visualized by adding 100 μl ABTS [2,2′-azino-bis(3-ethylbenzthiazoline-6-sulfonic acid); Sigma-Aldrich, St. Louis, MO]. After maintaining in the dark for 15 min, OD was measured at 405 nm with a microplate reader (EMax® Precision Microplate Reader, Molecular Devices, Biberach, Germany). Blanks were substracted.

### Photodynamic inactivation of bacteria

After a cultivation period of 72 h, biofilms were washed twice with sterile PBS to remove non-adherent bacteria and were incubated either with 50 μl of the respective PS at a concentration of 100 μM (experimental group PS+L+: *PIB*; control group PS+L-: *treatment with PS only*) or with 50 μl PBS (control group PS-L-: *untreated control*; control group PS-L+: *treatment with light only*) for 25 min in the dark. Then the samples were irradiated either for 600 s (SAPYR, MB) or for 181 s (MB) (groups PS+L+ and PS-L+) or maintained in the dark during the same period (groups PS+L- and PS-L-).

Immediately afterwards PS or PBS was carefully removed and each biofilm was brought to suspension with 200 μl of PBS by multiple up-and-down-pipetting and transferred to a 1.5 ml Eppendorf tube. These were placed in an ultrasonic water-bath chamber (35 kHz; Qualilab USR30H, Merck Eurolab, Bruchsal, Germany) for 5 min and then vigorously vortexed (REAX top, Heidolph Instruments, Schwabach, Germany) for 5 s for separation of aggregated bacteria. Serial tenfold dilutions (10^−2^ to 10^−7^) were prepared in BHI-broth and aliquots (3 × 20 μl) were plated on Mueller-Hinton-agar (*E. faecalis*) or blood agar (*A. naeslundii*) plates, respectively, according to the methodology described by Miles et al. (1938). Plates were incubated at 37°C for 24 h (*E. faecalis*) or 48 h (*A. naeslundii*). Afterwards, colony forming units (CFU) were counted.

### Data analysis

For ELLA experiments (data from 12 independent samples), OD at 405 nm was plotted versus the logarithm of Con A concentrations. Values were fitted in a dose-response curve including 95% confidence intervals using Table Curve 2D (Systat Software Inc., San Jose, CA).

All results of PIB experiments are graphically shown as medians, including 25 and 75% quantiles and were calculated using SPSS for Windows, version 20 (SPSS Inc., Chicago, IL) from the values of at least six independent experiments, each performed in duplicate. Horizontal solid and dashed lines in the figures depict reductions of 3 and 5 log_10_ steps CFU respectively, compared to untreated control groups PS-L-. Medians on these lines exhibit inactivation efficacy rates of 99.9% (3 log_10_) or 99.999% (5 log_10_). According to the infection control guidelines, this is declared as biologically relevant antimicrobial activity or disinfectant effect, respectively (Boyce and Pittet, 2002).

## Results

### Confirmation of extracellular polymeric substance (EPS)

**Figure 3:**
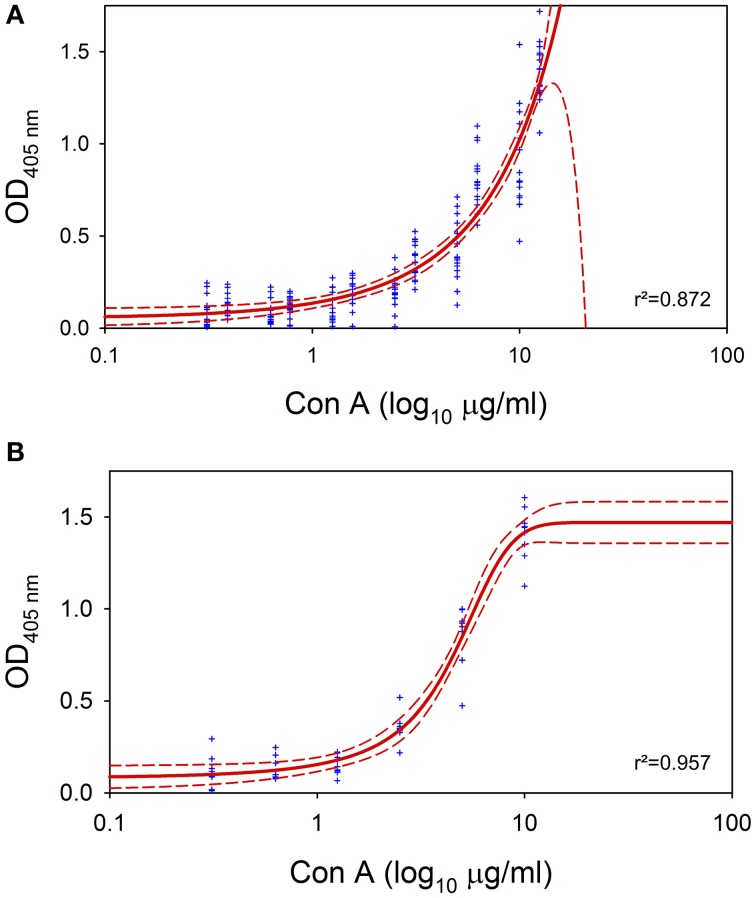
**Enzyme-linked lectinsorbent assay (ELLA)**. ELLA applied on 72 h *E. faecalis* and *A. naeslundii* monospecies biofilms for confirmation of EPS. Blue dots represent original measured values, red solid line represents the fit of the sigmoideal curve and red dashed lines depict 95% confidence intervals. r^2^ denotes the correlation coefficient. **(A)** ELLA on *E. faecalis* monospecies biofilm. **(B)** ELLA on *A. naeslundii* monospecies biofilm.

_D_-glucose and _D_-mannose residues in the EPS of *E. faecalis* and *A. naeslundii* monospecies biofilms were detected with ELLA using Con A as a lectin. Plotting the OD values at 405 nm versus the logarithm of the concentration of Con A added, resulted in dose-response curves for biofilms of *E. faecalis* (*r*^2^ = 0.872) and *A. naeslundii* (*r*^2^ = 0.957) (Figure 3). Consequently, increasing concentrations of Con A result in heightened OD values, which affirms the presence of binding sites for Con A and therefore the presence of EPS containing _D_-glucose and _D_-mannose residues. Non-specific binding of Con A to carbohydrates in CS medium can be neglected, since blank values did not show any dose-response curves (data not shown).

### Photodynamic inactivation of bacteria

**Figure 4:**
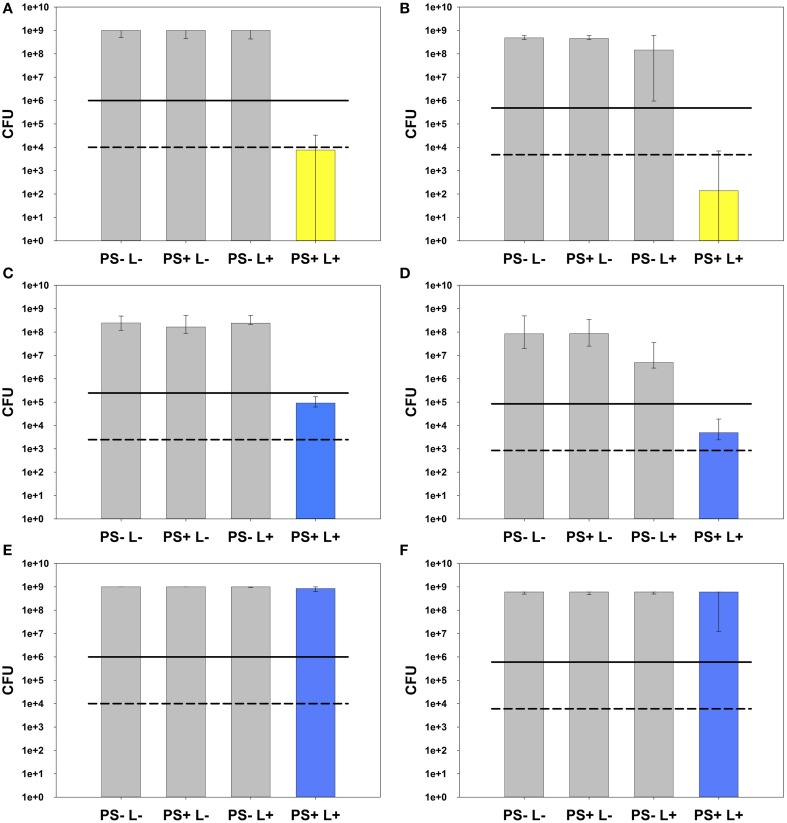
**Photodynamic inactivation of AN and EF monospecies biofilms**. All PIB results are shown as CFU medians with 25 and 75% quantiles depicted on a log_10_ scaled ordinate. Medians on or below the solid and dashed lines represent CFU reductions of ≥ 3 log_10_ and ≥ 5 log_10_ steps, respectively, compared to untreated control groups PS-L-. **(A)** PIB against *E. faecalis* monospecies biofilm using SAPYR (number of absorbed photons: 6.78 × 10^18^; energy dose: 30 J/cm^2^): PIB group PS+L+ (yellow) shows a reduction by 5.1 log_10_ steps CFU. **(B)** PIB against *A. naeslundii* monospecies biofilm using SAPYR (number of absorbed photons: 6.78 × 10^18^; energy dose: 30 J/cm^2^): PIB group PS+L+ (yellow) shows a reduction by 6.5 log_10_ steps CFU. PS-L+ group shows a reduction by 0.5 log_10_ steps CFU. **(C)** PIB against *E. faecalis* monospecies biofilm using MB (adjusted energy dose: 30 J/cm^2^; corresponding number of absorbed photons: 56.5 × 10^18^): PIB group PS+L+ (blue) shows a reduction by 3.4 log_10_ steps CFU. **(D)** PIB against *A. naeslundii* monospecies biofilm using MB (adjusted energy dose: 30 J/cm^2^; corresponding number of absorbed photons: 56.5 × 10^18^): PIB group PS+L+ (blue) shows a reduction by 4.2 log_10_ steps CFU. PS-L+ group shows a reduction by 1.2 log_10_ steps CFU. **(E)** PIB against *E. faecalis* monospecies biofilm using MB (adjusted number of absorbed photons: 6.78 × 10^18^; corresponding energy dose: 3.6 J/cm^2^): PIB group PS+L+ (blue) shows no reduction of CFU. **(F)** PIB against *A. naeslundii* monospecies biofilm using MB (adjusted number of absorbed photons: 6.78 × 10^18^; corresponding energy dose: 3.6 J/cm^2^): PIB group PS+L+ (blue) shows no reduction of CFU.

SAPYR and MB were evaluated against *E. faecalis* and *A. naeslundii* monospecies biofilms. Irradiation parameters for SAPYR were determined to irradiation with PIB 3000 for 600 s obtaining irradiance at sample-level of 50 mW/cm^2^, which corresponds to an energy dose of 30 J/cm^2^ and a number of 6.78 × 10^18^ absorbed photons. Under these conditions, SAPYR revealed an inactivation efficacy of 5.1 log_10_ steps against *E. faecalis* (Figure 4A) and 6.5 log_10_ steps against *A. naeslundii* (Figure 4B).

For comparing MB at adjusted energy dose compared to SAPYR (30 J/cm^2^; number of absorbed photons: 56.5 × 10^18^), MB was irradiated with PDT 1200L for 600 s at an irradiance of 50 mW/cm^2^ reaching the samples, too. Here, CFU of *E. faecalis* were reduced by 3.4 log_10_ steps (Figure 4C) and CFU of *A. naeslundii* by 4.2 log_10_ steps (Figure 4D).

In contrast, for comparing MB at adjusted numbers of absorbed photons compared to SAPYR (6.78 × 10^18^; energy dose: 3.6 J/cm^2^), MB was irradiated with PDT 1200L for 181 s at an irradiance at sample-level of 20 mW/cm^2^, which resulted in no reduction of CFU-median against both, *E. faecalis* (Figure 4E) and *A. naeslundii* (Figure 4F).

In all cases there was no reduction of CFU after treatment with PS only (PS+L−). Treatment with light only (PS−L+) had no effect on CFU of *E. faecalis.* In the case of *A. naeslundii*, CFU were diminished slightly by 0.5 log_10_ step when irradiated with Waldmann PIB 3000 (50 mW/cm^2^; 30 J/cm^2^) and by 1.2 log_10_ steps when irradiated with Waldmann PDT 1200L at 30 J/cm^2^. In contrast, irradiation with PDT 1200L at 3.6 J/cm^2^ had no effect on CFU of *A. naeslundii*.

## Discussion

Aim of the present study was to compare the antimicrobial photodynamic efficacy of given PS by using two strategies for adjustment of irradiation parameters. Since different PS exhibit distinct absorption characteristics, distinct light sources have to be used and their irradiation parameters have to be adjusted in order to ensure appropriate comparison of antimicrobial photodynamic efficacy. Usually, for adjustment of irradiation parameters only the energy doses applied by the respective light sources are matched. In this instance neither the molar extinction coefficients of a given PS nor the energy per wavelength emitted by the respective light source are factored in. Furthermore, the number of emitted photons of a given light source not only depends on the power but also on the wavelength of the emitted photons. In this study, a formula is presented considering all these aspects in order to calculate the numbers of photons, which are absorbed per second by a given PS when irradiated by its corresponding light source:

$$\begin{array}{l} \text{absorbed photons/ second}=\\ \;\sum_{\lambda }\left(1-10^{-\varepsilon (\lambda )\cdot \text{ c}\cdot \text{ d}}\right)\;\cdot \;{\text{P}}_{\text{em}}(\lambda )\;\cdot \;\frac{\lambda }{{\text{c}}_{0}\;\cdot \text{ h}} \end{array}$$

For a certain wavelength λ_0_, $\frac{{\text{c}}_{0}\;\cdot \text{ h}}{\lambda_{0}}$ describes the energy of a single photon. Its reciprocal $\frac{\lambda_{0}}{{\text{c}}_{0}\;\cdot \text{ h}}$ is multiplied by the spectral radiant power P_em_ (λ_0_) to obtain the number of emitted photons per second. The absorption of the PS is expressed by the factor (1−10^−ε (λ^_0_)·c ·d). The sum is calculated for wavelengths ranging from 300 to 800 nm in 1-nm-steps.

Depending on the mechanism of photodynamic action and based on the Jablonski diagram (Baier et al., 2007; Maisch et al., 2007), it is plausible that one PS molecule is excited by the absorption of one photon, whereby the ground state of the PS is transferred to its singlet state. By intersystem crossing the energy can change over to an excited sensitized triplet state, which can now generate singlet oxygen molecules.

So, adjusting the numbers of absorbed photons of distinct PS-light source systems ensures equal numbers of activated PS-molecules, thus allowing comparison of the antimicrobial photodynamic efficacy rates per excited PS-molecule. Therefore, distinct photodynamic antimicrobial efficacy despite adjusted numbers of absorbed photons may not be caused by photophysical properties but rather by PS-inherent features like mechanism of action (type I or type II; singlet oxygen quantum yield), attachment, uptake, intracellular localization etc.

For experimental validation of this formula, we exemplarily compared antimicrobial photodynamic efficacy rates of phenalen-1-one PS SAPYR to that of clinical standard PS MB (adjusted either by energy doses or by numbers of absorbed photons) regarding inactivation of monospecies biofilms formed by oral key pathogens *E. faecalis* and *A. naeslundii*. According to the general definition of a biofilm it is crucial to verify the presence of extracellular polymeric substance (EPS) before using the term “biofilm” for attached bacteria (Donlan and Costerton, 2002). With respect to the biofilms cultured according the protocol used in the present study, the presence of EPS has already been shown earlier by fluorescence microscopy using fluorescence-labeled Con A (Cieplik et al., 2013). Con A is a lectin that specifically binds to _D_-glucose and _D_-mannose residues, which are the saccharide components most frequently encountered in bacterial EPS (Sutherland, 1990). In this study an enzyme-linked lectinsorbent assay (ELLA) modified after the manual published by Leriche et al. (2000) was conducted, whereby peroxidase-labeled Con A was used as a lectin. Increasing concentrations of Con A resulted in heightened OD values whereby rendering clear dose-response curves, which demonstrates the existence of binding sites for Con A. Consequently the presence of EPS containing _D_-glucose and _D_-mannose residues was confirmed (Figure 3).

Irradiation parameters for SAPYR were designated and for irradiation of MB either the energy dose applied by the respective light sources or the numbers of absorbed photons were adjusted compared to SAPYR. For the latter, we inserted the constants, the experimental data and the measured values in the formula presented above in order to calculate the numbers of photons, which were absorbed per second by each PS when irradiated by its corresponding light source.

Under these conditions, SAPYR was able to inactivate monospecies biofilms of *E. faecalis* by 5.1 log_10_ steps of CFU, which is defined as a disinfectant effect (Boyce and Pittet, 2002). Biofilms of *A. naeslundii* could even be eradicated by 6.5 log_10_ steps. When comparing the inactivation efficacy of MB at adjusted energy dose, inactivation efficacy of MB was approximately 2 log_10_ steps inferior (*E. faecalis*: 3.4 log_10_; *A. naeslundii*: 4.2 log_10_). When evaluating MB at adjusted numbers of absorbed photons, MB exhibited no CFU-reduction at all. Due to this pronounced difference in the efficacy rates of MB according to whether energy dose was adjusted or the numbers of absorbed photons, adjusting the numbers of absorbed photons has to be considered in further studies since reliable comparison of given PS and corresponding light sources is essential for further optimization of PIB efficacy.

Despite adjusted numbers of absorbed photons, there were distinct antimicrobial photodynamic efficacy rates of SAPYR and MB in this study, which may be explained by PS-inherent aspects like mentioned above: Since there is no remarkable difference in molecular dimension between SAPYR and MB (272.3 and 284.4 g/mol without counterions, respectively), the extent of steric hindrance for penetration through EPS may be similar and unlikely accounts for the observed difference in the PIB efficacy. As both, SAPYR and MB, are single positively charged, electrostatic interactions with negatively charged EPS molecules should be similar, too. Though, it is known that SAPYR may act like a tenside due to its chemical structure comprising the combination of a hydrophilic pyridinium unit and a large hydrophobic tail (Cieplik et al., 2013). Consequently, this tenside-like character of SAPYR may facilitate disruption of the EPS and penetration of this PS throughout the biofilm, thus enhancing PIB efficacy. On the other hand, phenothiazinium salts like MB are known to be substrates for efflux pumps in a variety of bacterial species (Tegos and Hamblin, 2006), which may reduce antimicrobial photodynamic efficacy. However, this may be overcome by combined administration of MB with efflux pump inhibitors (Kishen et al., 2010).

Since ^1^O_2_ is known to be the main ROS in the mechanism of PIB (Maisch et al., 2007), the higher killing efficacy of SAPYR may also be attributed to its substantially higher ^1^O_2_ quantum yield compared to MB (0.99 vs. 0.52). Consequently, SAPYR generates considerably more ^1^O_2_ than MB, supporting its higher killing efficacy. In addition, MB is a metachromatic compound that forms dimers or higher aggregates at increasing PS concentrations, which tend to react mainly according to type I mechanism (Usacheva et al., 2003; Núñez et al., 2015).

In a former study, SAPYR was evaluated against monospecies and polyspecies biofilms formed by *E. faecalis*, *A. naeslundii*, and *Fusobacterium nucleatum* and demonstrated pronounced antimicrobial photodynamic efficacy rates (≥4 log_10_ steps of CFU reduction in the polyspecies biofilm) (Cieplik et al., 2013). The porphyrin derivative TMPyP, which was used as a control PS, had no effect at all, although it is described as efficient PS for inactivation of biofilms (Di Poto et al., 2009; Collins et al., 2010; Cieplik et al., 2014b). However, the emission of the light source, which was used in this study for irradiation of SAPYR and TMPyP (blue light emitting dental light-curing unit Bluephase C8, Ivoclar-Vivadent, Schaan, LIE; λ _em_ = 460 ± 20 nm), exhibited only partial overlap with the absorption of SAPYR. Consequently, the overall absorbed energy of TMPyP was about 6.4 times higher compared with the absorbed energy of SAPYR and the absolute yield for ^1^O_2_ generation was 4.8 times higher for TMPyP (Cieplik et al., 2013). In contrast, here a light source was employed with an emission spectrum adapted to the absorption of SAPYR (Waldmann PIB 3000). Likewise, for irradiation of MB a light source was chosen with an appropriate emission spectrum for the absorption of MB (Waldmann PDT 1200L).

When comparing the PIB efficacy rates of SAPYR here to those of the former study, *A. naeslundii* was inactivated more efficiently here (≥6 log_10_ compared to ≥ 2 log_10_), whereas inactivation rates against *EF* are identical (≥5 log_10_ both) (Cieplik et al., 2013). Since the concentration of SAPYR was identical in both studies (100 μM), the optimized emission spectrum of the Waldmann PIB 3000 may be causative for this. In addition, due to the better overlap of the emission of PIB 3000 with the absorption of SAPYR, the total treatment time for PIB with SAPYR could be reduced from 62 min (60 min incubation followed 2 min irradiation) to 35 min (25 min incubation followed by 10 min irradiation). Besides that, the irradiance at sample-level could be strikingly decreased from 600 mW/cm^2^ to 20 mW/cm^2^. This is an important aspect for clinical application, since high-power light-curing units (like Bluephase C8) may cause damage to oral soft tissues due to heat generation (Spranley et al., 2012).

Apart from that, it was found that irradiation of *A. naeslundii* with blue or red light at an energy dose of 30 J/cm^2^ without any PS led to a slight reduction of CFU (Waldmann PIB 3000: 0.5 log_10_; Waldmann PDT 1200L: 1.2 log_10_). This finding is in contrast to the former study, where no light toxicity was observed when *A. naeslundii* biofilms were irradiated with blue light derived from Bluephase C8 (Cieplik et al., 2013). However, this may be explained by different emission spectra of these light sources and a prolonged period of irradiation (600 s compared to 120 s) in the present study. An effect of heat can be excluded because this light toxicity could only be observed with monospecies biofilms of *A. naeslundii* but not *E. faecalis*. Therefore, the presence of endogenous substances in *A. naeslundii* (e.g. porphyrins) that can act as PS when irradiated with blue or red light may be accounted for that occurrence like it has already been demonstrated for some other oral pathogens like *Aggregatibacter actinomycetemcomitans* (Cieplik et al., 2014a) or *Porphyromonas gingivalis* and *Prevotella* spp. (Soukos et al., 2005). These endogenous PS may only be formed when *A. naeslundii* cells are in a sessile biofilm-state because no light toxicity could be observed when planktonic cultures of *A. naeslundii* were irradiated with PIB 3000 for the same period (data not shown). This is conceivable due the large amount of genetic diversity, which is generated when bacteria grow in a biofilm, in contrast to the homogenous populations of genetically identical cells, which are produced by growth of bacteria in planktonic cultures (Boles et al., 2004; Kolter and Greenberg, 2006). However, further studies are needed for understanding this auto-photosensitization process in sessile *A. naeslundii* cells.

Taken together, the results of this study provide evidence that PIB with SAPYR may be a promising approach for inactivation of biofilms, whereas PIB with MB is ineffective in this regard. However, before any clinical application of SAPYR, its toxicity against host cells has to be examined in subsequent studies. Furthermore, the capability of blue light to penetrate dental hard and gingival tissues has to be investigated for proper light-activation of SAPYR like it has already been shown for red light and MB (Ronay et al., 2013). In this regard, PIB with a combined administration of two PS (e.g. SAPYR and MB) might provide a synergistic strategy to enhance the antimicrobial photodynamic effect in more complex systems like the oral cavity (Acedo et al., 2014). In this instance irradiation with broadband light (≈400–650 nm) would be necessary for sufficient activation of both PS.

In general, a PS should feature high binding affinity for microorganisms (positive charge for adherence to negatively charged bacterial cell walls) (Alves et al., 2009) and low chemical toxicity and mutagenicity (Soukos and Goodson, 2011). In addition, photostability during irradiation is a critical point (no formation of toxic by-products upon irradiation) (Engel et al., 2008) and the respective combination of PS and light source has to be coordinated to yield an appropriate system for good PIB efficacy.

The data of this study clearly demonstrates that adjusting the numbers of absorbed photons is crucial for comparing antimicrobial photodynamic efficacy rates of distinct PS. This is essential for further development and/or optimization of both, PS and light sources.

## Conclusion

In this study, a formula is presented for calculation of the number of photons absorbed by a given PS upon irradiation by its corresponding light source. In this way, appropriate comparison of PIB efficacy of given PS-light source systems is ensured because according to the photodynamic principle one PS molecule can only be excited by the absorption of one photon.

For experimental validation of this formula, antimicrobial photodynamic efficacy of the phenalen-1-one PS SAPYR was exemplarily compared to that of clinical standard PS Methylene Blue regarding inactivation of monospecies biofilms formed by *E. faecalis* and *A. naeslundii*. PIB with SAPYR demonstrated a disinfectant efficacy on both pathogens whereas Methylene Blue exhibited a smaller effect when energy dose was adjusted compared to SAPYR. In contrast, when the number of absorbed photons was adjusted compared to SAPYR, no effect could be observed at all. Due to the pronounced differences of the photodynamic efficacy of MB when either energy dose or numbers of absorbed photons were adjusted compared to SAPYR, calculation of the numbers of absorbed photons has to be considered for further studies when the antimicrobial photodynamic efficacy rates of distinct PS are compared. Besides that, the high efficacy of photodynamic inactivation exhibited by SAPYR encourages further research on PIB with this PS as an approach for treatment and control of oral biofilm infections.

### Conflict of interest statement

The authors declare that the research was conducted in the absence of any commercial or financial relationships that could be construed as a potential conflict of interest.
